# Disease-related PSS1 mutant impedes the formation and function of osteoclasts

**DOI:** 10.1016/j.jlr.2023.100443

**Published:** 2023-09-14

**Authors:** Sari Sugahara, Yuki Ishino, Koki Sawada, Tsumugi Iwata, Yuta Shimanaka, Junken Aoki, Hiroyuki Arai, Nozomu Kono

**Affiliations:** Department of Health Chemistry, Graduate School of Pharmaceutical Sciences, The University of Tokyo, Tokyo, Japan

**Keywords:** Glycerophospholipids, Phospholipids, Phospholipids/Biosynthesis, Phospholipids/Metabolism, Lipidomics, phosphatidylserine, osteoclasts, Lenz-Majewski syndrome

## Abstract

Phosphatidylserine (PS) is an acidic phospholipid that is involved in various cellular events. Heterologous dominant mutations have been identified in the gene encoding PS synthase 1 (PSS1) in patients with a congenital disease called Lenz-Majewski syndrome (LMS). Patients with LMS show various symptoms, including craniofacial/distal-limb bone dysplasia and progressive hyperostosis. The LMS-causing gain-of-function mutants of PSS1 (PSS1^LMS^) have been shown to synthesize PS without control, but why the uncontrolled synthesis would lead to LMS is unknown. Here we investigated the effect of PSS1^LMS^ on osteoclasts (OCs) to elucidate the causative mechanism of LMS. PSS1^LMS^ did not affect the expression of OC-related genes but inhibited the formation, multinucleation, and activity of OCs. Especially, OCs expressing PSS1^LMS^ showed abnormal patterns and dynamics of actin podosome clusters, which have roles in OC migration and fusion. PSS1^LMS^ did not affect the level of PS but changed the acyl chain compositions of PS and phosphatidylethanolamine, and decreased the level of phosphatidylinositol. The introduction of a catalytically inactive mutation into PSS^LMS^ canceled the changes in phospholipids and the phenotypes observed in OCs expressing PSS1^LMS^. A gain-of-function mutant of PSS2 (PSS2 R97K) also impaired OC formation and caused changes in phospholipid composition similar to the changes caused by PSS1^LMS^. Our results suggest that uncontrolled PS synthesis by PSS1^LMS^ causes changes in the quantity or fatty acid composition of certain phospholipid classes, impairing OC formation and function, which might be a cause of osteosclerosis in patients with LMS.

Phosphatidylserine (PS) is a major phospholipid class constituting cell membranes. PS has a negative charge in its polar head group, through which PS interacts with proteins in various biological processes (1, 2). PS is synthesized by two PS synthases, PSS1 and PSS2 (3, 4). They are both membrane proteins and have 38% sequence homology. PSS1 and PSS2 use phosphatidylcholine (PC) and phosphatidylethanolamine (PE) as substrates, respectively, and introduce a serine moiety to the head group by a base-exchange reaction. The activity of PSSs is known to be inhibited by their product PS. PS synthesis is suppressed when PS is exogenously added to cells (5, 6, 7). Mice deficient in PSS1 or PSS2 have no significant change in phospholipid composition (except for liver in PSS1-deficient mice) and do not show any noticeable phenotype, while PSS1 and PSS2 double-deficient mice are embryonic lethal (8, 9).

Several mutations in the PSS1-encoding gene, *PTDSS1*, were found in patients with a congenital disease called Lenz-Majewski syndrome (LMS) (10). LMS is a sporadic disease, with only 20 patients reported so far (11). The patients show various symptoms like craniofacial/distal-limb bone dysplasia, progressive hyperostosis, cutis laxa, and intellectual disability. An analysis of the whole-exome sequence identified three mutations in *PTDSS1* in five unrelated patients (10). That report and subsequent studies (11, 12, 13, 14) have identified a total of seven missense mutations at six amino acid residues. The PSS1 mutants (hereafter referred to as PSS1^LMS^) showed elevated PS synthetic activity, accompanied by elevated PE formation (10), which is probably due to the conversion of PS to PE by PS decarboxylase PISD (15). Interestingly, fibroblasts from patients with LMS were resistant to the feedback inhibition of PS synthetic activity by exogenous PS, indicating the elevated PS synthetic activity of PSS1^LMS^ is due to the loss of feedback inhibition (10). However, the amount of cellular PS, PC, and PE did not change in fibroblasts from patients with LMS (10). Therefore, it is still unknown why uncontrolled PS synthesis causes LMS.

The most striking symptoms of LMS are bone dysplasia and sclerosis. In clinical reports, many patients showed craniofacial osteosclerosis, which could result in nasal airway obstruction or hydrocephalus by blocking cerebrospinal fluid at the sclerosing craniovertebral junction (12, 16). Nasal airway obstruction can induce apneic spells, which was the cause of death in one reported patient (16). Hydrocephalus, which also inhibits normal mental development, was alleviated by surgery at the craniovertebral junction in the same patient (16). Thus, it is considered important to understand the mechanism of osteosclerosis caused by uncontrolled PS synthesis.

Osteoclasts (OCs) are the main cells involved in destroying bone. Impairment of OC function results in osteopetrosis or osteosclerosis (17, 18). The bone density in these patients increases, and the bone usually becomes hard but fragile. Osteopetrosis can cause anemia due to smaller bone marrow or impair breathing, vision, hearing, and functioning of the central nervous system (18). The latter symptoms are often caused by narrower cavities in the skull resulting from craniofacial sclerosis, which is similar to what was observed in patients with LMS. Based on this similarity between patients with LMS and patients with osteopetrosis, we investigated whether uncontrolled PS synthesis affects OC formation or function.

## Materials and Methods

### Materials

L-Serine, [^14^C(U)] (MC-265) was purchased from Moravek, Inc. Brain PS was purchased from Sigma-Aldrich. Recombinant murine macrophage colony-stimulating factor (MCSF) (315-02) was purchased from PeproTech. Glycerol, [^14^C(U)] (NEC441X) was purchased from PerkinElmer. 12:0/13:0 PC, PE, PS, and phosphatidylinositol (PI) were from Avanti Polar Lipids.

### Mice

Mice were housed in climate-controlled (23°C) pathogen-free facilities with a 12-h light-dark cycle, with free access to standard chow (CE2; CLEA Japan) and water. All animal experiments were performed in accordance with protocols approved by the Animal Committees of the University of Tokyo in accordance with the Standards Relating to the Care and Management of Experimental Animals in Japan.

### Antibodies

Antibodies to PSS1 (ab157222) and β-actin (ab8226) were purchased from Abcam. Antibody to GAPDH (6C5) was purchased from Calbiochem, Merck Millipore. Antibodies to Vinculin (v9131), Talin (t3287), and α-tubulin (DM1A) were purchased from Sigma-Aldrich. Antibodies to Src (#2110), pSrc (Y416) (#2101), phospholipase C γ2 (PLCγ2) (#3872), and phosphorylated PLCγ2 (Y1217) (#3871) were purchased from Cell Signaling Technology, Inc. Antibody to Paxillin was purchased from BD Biosciences. Antibodies to FLAG (1E6) and His tag (9F2) were purchased from FUJIFILM Wako Pure Chemical Co.

### Plasmids

Mouse *Ptdss1* (NM_008959) and *Ptdss2* (NM_013782) were amplified from the cDNA of the mouse brain and cloned into pEGFP-N2. Then, mouse *Ptdss1-egfp* and *Ptdss2-egfp* were amplified and subcloned into pMXs-IP. Mutations were introduced by inverse PCR. pMXs-IP and pMXs-IB were kind gifts from Dr. T. Kitamura (the Univ. of Tokyo, Tokyo, Japan). *Lifeact-FusionRed* was incorporated into pMXs-IP. *Glutathione S-transferase* (*GST*)-mouse *receptor activator of nuclear* *factor-kappa* *B ligand* (m*RANKL*) in pGEX5X-2 was a generous gift from Dr. H. Suzuki (the Univ. of Tokyo, Tokyo, Japan). Mouse *Ptdss1-egfp-P2A-PuroR* and *Ptdss2-egfp-P2A-PuroR* were cloned into the backbone vector of lentiCRISPR v2 (addgene#52961) in replacement of the insert section from *U6* promoter, *Cas9* to *PuroR*. *FLAG*-human *PTDSS1*-*HA* in pCMV (NM_014754) was a kind gift from Dr O. Kuge (Kyusyu Univ., Fukuoka, Japan). *FLAG*-human *PTDSS1-HA* was amplified and cloned into pMXs-IB. pSpCas9 (BB)−2A-Puro (PX459) vector was from Addgene (addgene#48139).

### Cell culture

RAW264.7 cells were cultured with α-MEM (Sigma-Aldrich) supplemented with 10% FBS (Gibco) and Penicillin-Streptomycin-Glutamine (PSG) (Gibco). HeLa, HEK293T, and PlatE cells were cultured with DMEM (Sigma-Aldrich) supplemented with 10% FBS and PSG. To culture PlatE cells, blasticidin S (Kaken Pharmaceutical Co., Ltd.) and puromycin (FUJIFILM Wako Pure Chemical Co.) were added at the final concentration of 10 μg/ml and 2 μg/ml, respectively. RAW264.7 cell line was a kind gift from Dr. H. Suzuki (the Univ. of Tokyo, Tokyo, Japan). HeLa and HEK293T cell lines were obtained from ATCC. PlatE cell line was a generous gift from Dr. T. Kitamura (the Institute of Med. Sci., the Univ. of Tokyo, Tokyo, Japan).

### Preparation of recombinant GST-mRANKL

GST-mRANKL was expressed in *E. coli.* Rosetta-gami 2 cells (Novagen, Sigma-Aldrich). Bacterial cultures in Luria-Bertani (LB) medium were induced with 20 μM isopropyl β-D-1-thiogalactopyranoside (FUJIFILM Wako Pure Chemical Co.) and grown for 20–22 h at 20°C. The bacterial pellet was resuspended in PBS and sonicated. The protein was purified using a GSTrap HP column and AKTA pure 25 (GE Healthcare Life Sciences) according to the manufacturer’s instructions. The fractions containing GST-mRANKL were confirmed with SDS-PAGE and concentrated to 0.5 mg/ml in PBS by ultrafiltration using Vivaspin (Sartorius).

### In vitro generation of OCs

Bone marrow cells (BMCs) of tibias and femurs from C57BL6/J wildtype mice (male, 6–8 weeks old from CLEA Japan, Inc.) were flushed out with α-MEM (PSG). The cells were collected by centrifugation at 300 g for 5 min, resuspended with α-MEM (10%FBS, PSG), and filtrated with a 70 μm cell strainer (BD Biosciences). MCSF was added at the final concentration of 10 ng/ml and the cells were cultured overnight (17–18 h) in 10 cm Petri dishes at 37°C, 5% CO_2_. On the next day, floating cells were collected by centrifugation at 300 g for 5 min. Cells were counted and seeded at 1.5 × 10^5^ cells/cm^2^ with MCSF (50 ng/ml) and GST-RANKL (100 ng/ml) (day 0). The medium was changed every 2 days and OCs formed from days 4 to 6.

### Preparation of retrovirus for protein expression in OCs

pMXs-IP encoding the desired protein was introduced into PlatE cells with Lipofectamine LTX (Invitrogen) following the manufacturer’s instructions. After 4–6 h from transfection, the medium was changed to α-MEM (10% FBS, PSG), and the cells were cultured for another 2 days. Then the medium was collected and centrifuged at 3,000 rpm for 10 min, followed by filtration with a 0.45-μm filter (Merck Millipore). The resulting suspension was used as a retrovirus solution.

### Retrovirus treatment of OCs

At day 0 of in vitro generation of osteoclasts, the cells were seeded at 3 × 10^5^ cells/cm^2^ with MCSF and RANKL. On day 1, the medium was changed to retrovirus solution supplemented with MCSF (50 ng/ml) and RANKL (100 ng/ml) and incubated at 37°C, 5% CO_2_ for 4–6 h. Then cells were washed once with PBS and cultured again in α-MEM (10% FBS, PSG) with MCSF and RANKL. The medium was changed every 2 days and OCs formed from days 4 to 7.

### OC generation from RAW264.7 cells

RAW264.7 cells were seeded at 1.5 × 10^3^ cells/cm^2^. On the next day, 10 ng/ml MCSF and 100 ng/ml GST-mRANKL were added by changing the medium. The medium was changed every two days and OCs formed in 4–5 days.

### Generation of RAW264.7 cell lines expressing PSS1-GFP

HEK293T cells were transfected with the lentiviral vector containing PSS1-GFP-P2A-PuroR, psPAX2, and pMD2.G. using Lipofectamine LTX (Invitrogen), according to the manufacturer’s protocol. The medium was changed to α-MEM (10% FBS, PSG) 4–6 h after transfection, and the cells were cultured for another 2 days. RAW264.7 cells were seeded in 10-cm dishes. On the next day, lentivirus suspension was collected and added to RAW264.7 cells. The cells were incubated with lentivirus for 4–6 h, and the medium was changed. The cells were selected with α-MEM (10% FBS, PSG) containing 4 μg/ml puromycin. The puromycin-selected cells were sorted by PSS1-GFP expression level using a cell sorter SH800 (SONY).

### Generation of PSS1 knockout HeLa cells

The single guide RNA (sgRNA) sequence targeting the first exon of *PTDSS1* was designed by CRISPRdirect (19). The sgRNA sequence (5′-tcatgtacttcgcctttacc-3′) was then cloned into the pSpCas9 (BB)−2A-Puro (PX459) vector. The PX459 vector containing human *PTDSS1* sgRNA was transfected to HeLa cells using Lipofectamine LTX, according to the manufacturer’s protocol. Twenty-four hours after transfection, the cells were selected with fresh medium containing 1 μg/ml puromycin. Seventy-two hours after selection with puromycin, cells were seeded as single cell clones by limiting dilution to generate individual knockout clones. The clones were then screened by western blotting with anti-PSS1 antibody.

### Preparation of PS liposomes

PS in chloroform was dried with nitrogen gas. Autoclaved PBS was added to the dried PS to give the final concentration of 5 mM PS and incubated for 1 h at room temperature, under protection from light. To generate PS liposomes, the solution was vortexed for 30 s three times and sonicated with a bath sonicator at 4°C for 30–40 min. The liposome solution was diluted in culture medium and added to cells.

### The PS synthesis assay with thin layer chromatography using [^14^C]serine

The OCs expressing PSS1 were selected by adding 2 μg/ml puromycin to the medium. At day 7, when mature OCs formed, the OCs were labeled with [^14^C]serine (0.5 μCi/ml) for 3 h. For PS treatment, OCs were treated with 20 μM PS 2 h before and during [^14^C]serine labeling. Phospholipids were extracted from cells by the method of Bligh and Dyer (20). Then the extract was applied to thin layer chromatography and eluted with chloroform: methyl acetate: 1-propanol: methanol: 0.25% KCl = 25: 25 : 25: 10: 9. The radioactivity was detected with imaging plates and Typhoon FLA 9000 (GE Healthcare Life Sciences) and normalized to total protein content.

### Tartrate-resistant acid phosphatase staining

OCs were stained with a tartrate-resistant acid phosphatase (TRAP) staining kit (387A; Sigma-Aldrich) according to the manufacturer’s protocol. TRAP-positive cells were imaged with an EVOS FL Auto 2 Imaging System (Thermo Fisher Scientific) and counted manually using ImageJ.

### Assay for extracellular acidification activity

BMCs were seeded in Osteo Assay Surface multiple well plates (Corning, Inc.) at 0.2 × 10^6^ cells/well at day 0. On day 1, the cells were treated with retrovirus to express PSS1. The medium was changed every 2 days until day 7. On day 5, 0.85 μl of concentrated HCl was added to every 1 ml of medium to give the pH around 7 to promote activity (21). On day 7, 0.5% sodium hypochlorite was added and incubated at room temperature for 5 min to wash out cells. After washing three times with ultra-pure water, the wells were dried, and the whole well area was imaged with the EVOS FL Auto 2 Imaging System. The total size of degraded pits was measured with ImageJ.

### Quantitative RT-PCR

Total RNA was extracted from cells with ISOGEN II (Nippon Gene) and reverse transcribed with High-Capacity cDNA Reverse Transcription Kit (Applied Biosystems, Thermo Fisher Scientific) according to the manufacturer’s protocol. Quantitative RT-PCR analysis was performed with a LightCycler 480 II (Roche) or LightCycler 96 (Roche), using SYBR Premix Ex Taq II (Takara Bio) or KAPA SYBR FAST One-Step qRT-PCR Kit (NIPPON Genetics Co., Ltd). The primers used in RT-PCR are listed below.

mouse *Nfatc1* fwd: tccaaagtcattttcgtgga, rvs: ctttgcttccatctcccaga.

mouse *Acp5* fwd: cgtctctgcacagattgcat, rvs: aagcgcaaacggtagtaagg.

mouse *Ctsk* fwd: cgaaaagagcctagcgaaca, rvs: tgggtagcagcagaaacttg.

mouse *Dcstamp* fwd: cgaagctccttgagaaacga, rvs: ggactggaaaccagaaatgaa.

mouse *Atp6v0d2* fwd: aagcctttgtttgacgctgt, rvs: gccagcacattcatctgtacc.

mouse *Gapdh* fwd: aggtcggtgtgaacggatttg, rvs: tgtagaccatgtagttgaggtca.

mouse *Actb* fwd: atgaagatcaagatcattgctcctc, rvs: tgtccaccttccagcagatgt

### Microarray analysis

Total RNA was extracted from cells with ISOGEN II (Nippon Gene) at days 3 and 6. Cells from four independent wells were pooled to generate one sample of total RNA. The extracted RNA was purified with a High Pure RNA Isolation kit (Roche), and a microarray analysis was conducted by Clarion S Assay, mouse (Thermo Fisher Scientific). The resulting data were analyzed with Transcriptome Analysis Console software (Thermo Fisher Scientific). The microarray data can be accessed at the Gene Expression Omnibus (GEO) repository (GSE207351).

### Immunocytochemistry

BMCs were seeded on glass coverslips, which were pre-coated with collagen type I (Corning, Inc.) according to the manufacturer’s protocol. On day 1, the cells were treated with retrovirus to express PSS1-GFP. Generally, cells became mature OCs on day 4 and were fixed with 4% paraformaldehyde (FUJIFILM Wako Pure Chemical Co.) in PBS for 15 min at room temperature. After a three-time wash with PBS, cells were permeabilized with 0.1% Triton X-100 (FUJIFILM Wako Pure Chemical Co.) in PBS for 5 min at room temperature. The cells were washed again and then blocked with 3% bovine albumin serum (BSA) (Sigma-Aldrich) in PBS. The primary antibodies were diluted in 3% BSA-PBS and incubated with the cells overnight at 4°C. On the next day, the cells were incubated with secondary antibodies, fluorescent phalloidin (Invitrogen), or DAPI (Sigma-Aldrich) for 1 h at room temperature and mounted with PermaFluor (Lab Vision, Thermo Fisher Scientific). For 2xPH (evt2)-His staining, the cells were permeabilized with 50 μg/ml digitonin in PBS for 5 min at room temperature. 2xPH (evt2)-His was incubated together with anti-His primary antibody. Expression and purification of recombinant protein 2xPH (evt2)-His was described previously (22).

### Confocal microscopy

Confocal microscopy was performed using a TCS SP8 (Leica) with a 63 × 1.2 Plan-Apochromat water immersion lens.

### Live imaging

BMCs were seeded in IWAKI 35 mm glass base dishes (AGC Techno Glass, Shizuoka, Japan), which were precoated with collagen type I. The cells were treated with retrovirus to express PSS1^LMS^-GFP or fluorescent Lifeact. They became mature OCs on day 4, and live imaging was conducted. The cells were imaged in xyt mode at 37°C with 5% CO_2_ using a TCS SP8.

### Western blotting

OCs were collected with lysis buffer (20 mM Tris-HCl pH 7.4, 100 mM NaCl, 5 mM EDTA, 1% Triton X-100, 0.1% SDS). After incubation at 4°C for 20 min, the lysate was centrifuged at 12,000 rpm at 4°C to remove the insoluble fraction. The supernatant was used as the protein extract. Protein concentrations were determined by the BCA assay (Pierce). Proteins were separated by SDS-PAGE and transferred to PVDF membranes. The membranes were blocked with 5% BSA in TTBS buffer (10 mM Tris-HCl pH 7.4, 150 mM NaCl, 0.1% Tween 20) and incubated with primary antibodies overnight at 4°C. On the next day, the membranes were incubated with horseradish peroxidase-conjugated anti-mouse or anti-rabbit IgG antibody (GE Healthcare). The proteins were detected by enhanced chemiluminescence (ECL Western blotting detection system, GE Healthcare) using LAS4000 (GE Healthcare Life Sciences).

### MS analysis of phospholipids

Phospholipids were extracted from cells by the method of Bligh and Dyer (20). When OCs were treated with PS liposomes, the cells were washed with α-MEM (10% FBS, PSG) three times to remove lipids attaching to the outer leaflet of the cell membrane. LC-MS/MS-based lipidomic analyses were performed using a Shimadzu Nexera ultra-high-performance liquid chromatography system (Shimadzu) coupled with a QTRAP4500 hybrid triple quadrupole linear ion trap mass spectrometer (AB SCIEX), as described previously (23). 12:0/13:0 PC, PE, PI, and PS were used as internal standards.

### Phosphoinositides extraction, derivatization, and MS analysis

These procedures were conducted as described previously (24). The OCs expressing PSS1 were selected by adding 2 μg/ml puromycin (FUJIFILM Wako Pure Chemical Co.) to the medium. Samples were collected on day 7.

### Fluorescence after photobleaching

OCs were prepared in the same way they were prepared for live imaging. They were photobleached with 100% laser power for 2 s. Then, images were collected every 0.582 s for 1–2 min.

### [^14^C]Glycerol and [^32^P]orthophosphate labeling of phospholipids in PSS1-expressing HeLa cells

HeLa cells were infected with retrovirus solutions to express FLAG-human PSS1^WT^-HA or FLAG-human PSS1^LMS^-HA and selected with 10 μg/ml blasticidin S. HeLa cells were seeded in 6-well plates. To analyze de novo synthesis of phospholipids, the cells were labeled with 1.0 μCi [^14^C]glycerol or 10 μCi [^32^P]orthophosphate per well for 2 or 4 h, respectively, on the next day. Phospholipids were extracted by the method of Bligh and Dyer. The extract was applied to thin layer chromatography and eluted with chloroform: methyl acetate: 1-propanol : methanol: 0.25% KCl = 25: 25 : 25: 10: 9. The radioactivity was detected with imaging plates and Typhoon FLA 9000 (GE Healthcare Life Sciences) and normalized by total protein content.

To analyze the degradation of phospholipids, the cells were labeled with 0.3 μCi [^14^C]glycerol per well for 12 h on the day after seeding. Then the medium was washed out, and the radioactive label was chased for up to 0, 2, 8, or 24 h. Phospholipids analysis was done as described above.

## Results

### PSS1^LMS^ impairs the formation and function of OCs

To examine the effects of PSS1^LMS^ on OCs, we generated OCs in vitro from mouse bone marrow cells (BMCs) (25). Since the PSS1 mutations in LMS patients are heterologous dominant, BMCs from wild-type mice were infected with retrovirus to express GFP-tagged wildtype PSS1 (PSS1^WT^) or PSS1^LMS^. In this study, the Q353R mutant of PSS1, which was found in three of ten LMS patients, was used as PSS1^LMS^.

**Fig. 1:**
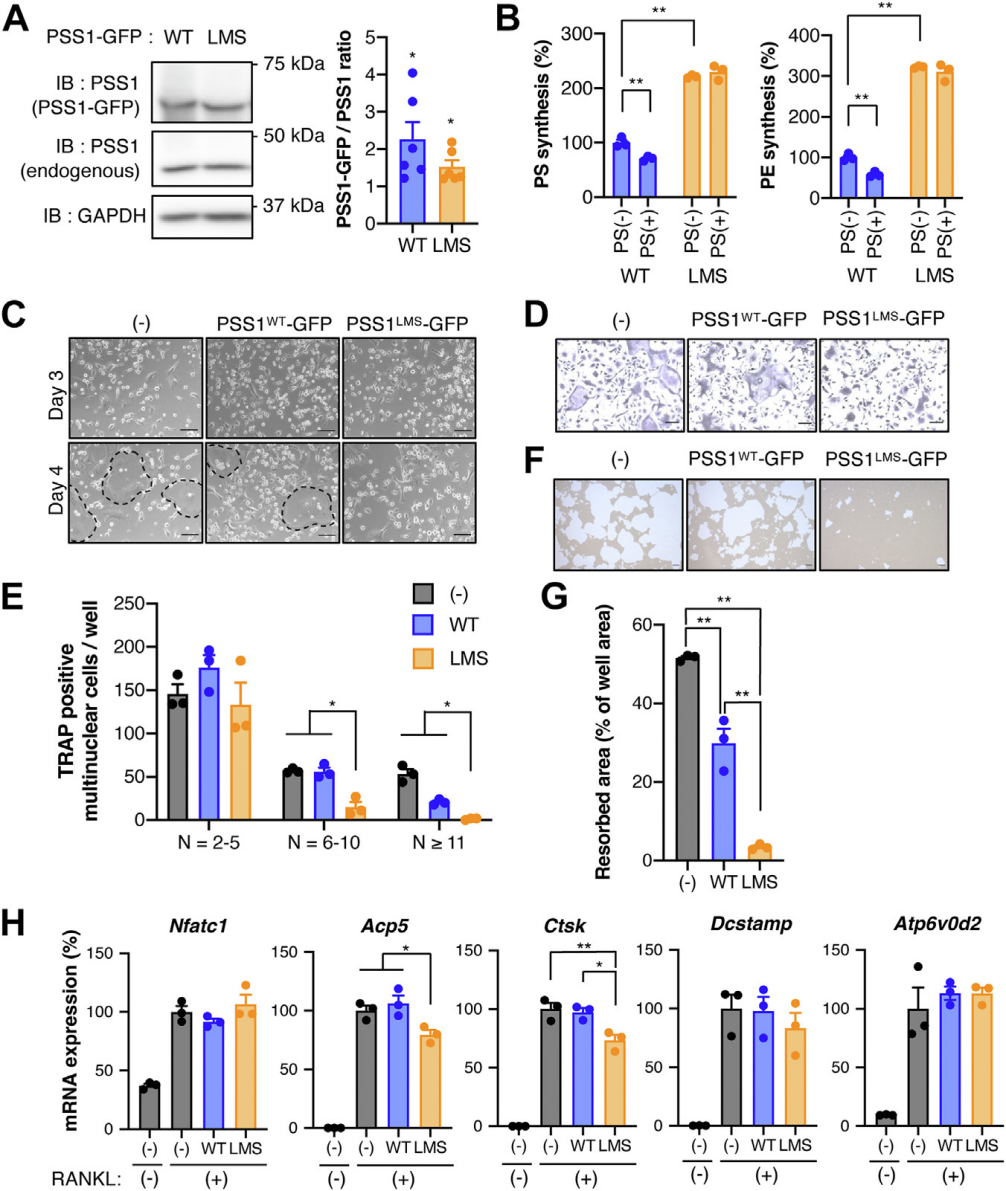
**PSS1**^**LMS**^**impairs the formation and function of osteoclasts.** A: PSS1^WT^- or PSS1^LMS^-GFP was expressed in OCs, and OCs were selected by puromycin. The amount of PSS1 was analyzed by immunoblotting. GAPDH was used as a loading control. The image is representative of six independent experiments. Data are Mean + SEM (n = 6). ∗*P* < 0.05 (one sample *t* test with theoretical mean of 1). B: PS and PE synthesis in PSS1^WT^- or PSS1^LMS^- expressing OCs were measured by [^14^C]serine incorporation, with or without exogenous 20 μM brain PS. Data are Mean + SEM (n = 3). C: Non-transduced (hereafter shown as (−) in figures), PSS1^WT^- or PSS1^LMS^-expressing OCs were observed by phase-contrast microscopy at day 3 or 4 after RANKL treatment. Dot lines indicate mature OCs. Scale bars, 100 μm. D: Non-transduced, PSS1^WT^- or PSS1^LMS^-expressing OCs were detected by TRAP staining. Scale bars, 100 μm. E: TRAP-positive multinucleated OCs in (D) were counted. N, number of nucleus per cell. Data are Mean ± SEM (n = 3). ∗*P* < 0.05. Statistical analysis was done by one-way ANOVA with Tukey’s test. F: Degraded pits formed by non-transduced, PSS1^WT^- or PSS1^LMS^-expressing cells were imaged. Scale bars, 100 μm. G: The degraded area in (F) was measured by ImageJ. Data are Mean ± SEM (n = 3). ∗∗*P* < 0.01. Statistical analysis was done by one-way ANOVA with Tukey’s test. H: The mRNA expression of the representatives of OC genes was analyzed by qRT-PCR. Data are Mean + SEM (n = 3). ∗*P* < 0.05; ∗∗*P* < 0.01. Statistical analysis was done by one-way ANOVA with Tukey’s test.

First, PS and PE synthetic activity were analyzed in OCs expressing PSS1^WT^ or PSS1^LMS^. In these cells, the amount of exogenously-expressed PSS1 was 1.5- to 2-fold greater than the amount of endogenous PSS1 on average (Fig. 1A). Although PSS1 has a molecular weight of 56 kDa, it appeared at about 40 kDa in SDS-PAGE, which is consistent with the previous study (26) and further confirmed by immunoblotting of PSS1 in PSS1 KO HeLa cells (supplemental Fig S1). [^14^C]Serine incorporation into PS and PE was detected in OCs with PSS1^WT^, which was inhibited by adding 20 μM PS to the culture medium (Fig. 1B). On the other hand, OCs with PSS1^LMS^ showed elevated PS synthesis both with and without PS. A similar increase was observed in PE synthesis.

When we observed OC formation with a phase-contrast microscope on day 3, there was no difference in the appearance of non-transduced, PSS1^WT^- or PSS1^LMS^-expressing cells. However, on day 4, non-transduced and PSS1^WT^-expressing cells became multinuclear OCs, whereas PSS1^LMS^-expressing cells did not (Fig. 1C). We also confirmed the formation of multinuclear OCs by TRAP (Tartrate Resistant Acid Phosphatase) staining in non-transduced or PSS1^WT^-expressing cells. On the other hand, PSS1^LMS^ expression resulted in reduced multinuclear OC formation (Fig. 1D, E).

To examine if PSS1^LMS^ affects the function of OCs, we analyzed the extracellular acidification activity of OCs by culturing them in calcium phosphate-coated wellplates and measuring the area where calcium phosphate was degraded. PSS1^LMS^-expressing cells showed markedly lower acidification activity than non-transduced or PSS1^WT^-expressing cells (Fig. 1F, G). PSS1^WT^-expressing cells also showed a decrease in acidification activity, but only by about 60% compared to non-transduced cells. These results suggest that the multinucleation and activity of OCs are impaired by PSS1^LMS^.

### PSS1^LMS^ does not affect gene expression during OC differentiation

Treatment of OCs with the cytokine RANKL induces the expression of genes that are crucial for OC fusion, multinucleation, and bone-resorbing activity. To examine whether PSS1^LMS^ affects gene expression, we analyzed the expressions of representative OC marker genes in PSS1^LMS^-expressing cells by quantitative real-time PCR (qRT-PCR). Expressions of the marker genes, such as *Nfatc1*, *Acp5*, *Ctsk*, *Dcstamp*, and *Atp6v0d2*, were unaffected or decreased only modestly compared with non-transduced or PSS1^WT^-expressing cells (Fig. 1H). We also conducted a microarray analysis to compare the gene expression profiles comprehensively. As expected, RANKL-untreated and RANKL-treated cells showed significantly different gene expression patterns. However, PSS1^WT^- and PSS1^LMS^-expressing cells showed almost the same gene expression profile (supplemental Fig S2).

In the experiments with mouse BMCs, RANKL treatment started before retrovirus infection. To see if PSS1^LMS^ does not affect gene expression profile even when expressed before RANKL treatment, we used RAW264.7 cultured cells which are macrophage-like cells that differentiate into OCs upon RANKL treatment (27). PSS1^WT^- or PSS1^LMS^-GFP-P2A-PuroR was stably expressed in RAW264.7 cells using lentivirus. In western blotting with anti-PSS1 antibody, the expressed proteins were detected only at the estimated molecular weight of PSS1-GFP and not at that of PSS1-GFP-P2A-PuroR (estimated at about 105 kDa), which indicates that the protein was efficiently processed at the P2A site (supplemental Fig S3A). When these cells were treated with RANKL and differentiated into OCs, TRAP-positive multinucleated cells and extracellular acidification activity were both reduced in PSS1^LMS^-expressing RAW264.7 cells (supplemental Fig S3B, C). On the other hand, the expressions of OC genes in PSS1^LMS^-expressing RAW264.7 cells were similar or only modestly decreased compared to their expressions in PSS1^WT^-expressing cells (supplemental Fig S3D), which supports the former results with BMCs. Collectively, these results indicate that PSS1^LMS^ does not significantly alter the gene expression profile in OCs.

### PSS1^LMS^-expressing OCs show abnormal patterns of the actin cytoskeleton

The actin cytoskeleton of OCs changes dynamically through differentiation, multinucleation, and bone resorption (28, 29). F-actin in OCs forms structures called podosomes, which are observed in limited cell types such as OCs, dendritic cells, and cancer cells. Each podosome consists of a core and cloud. The core is a dense F-actin structure, while the cloud is made of sparser F-actin. OCs form several clusters of podosomes when they differentiate. The podosomes then start to form a ring that expands and evolves into a podosome belt (29). Podosome belts are considered to be similar to sealing zones which OCs use to tightly attach to bone to resorb it (30). Podosome clusters and rings also have roles in OC migration and fusion (29, 31).

**Fig. 2:**
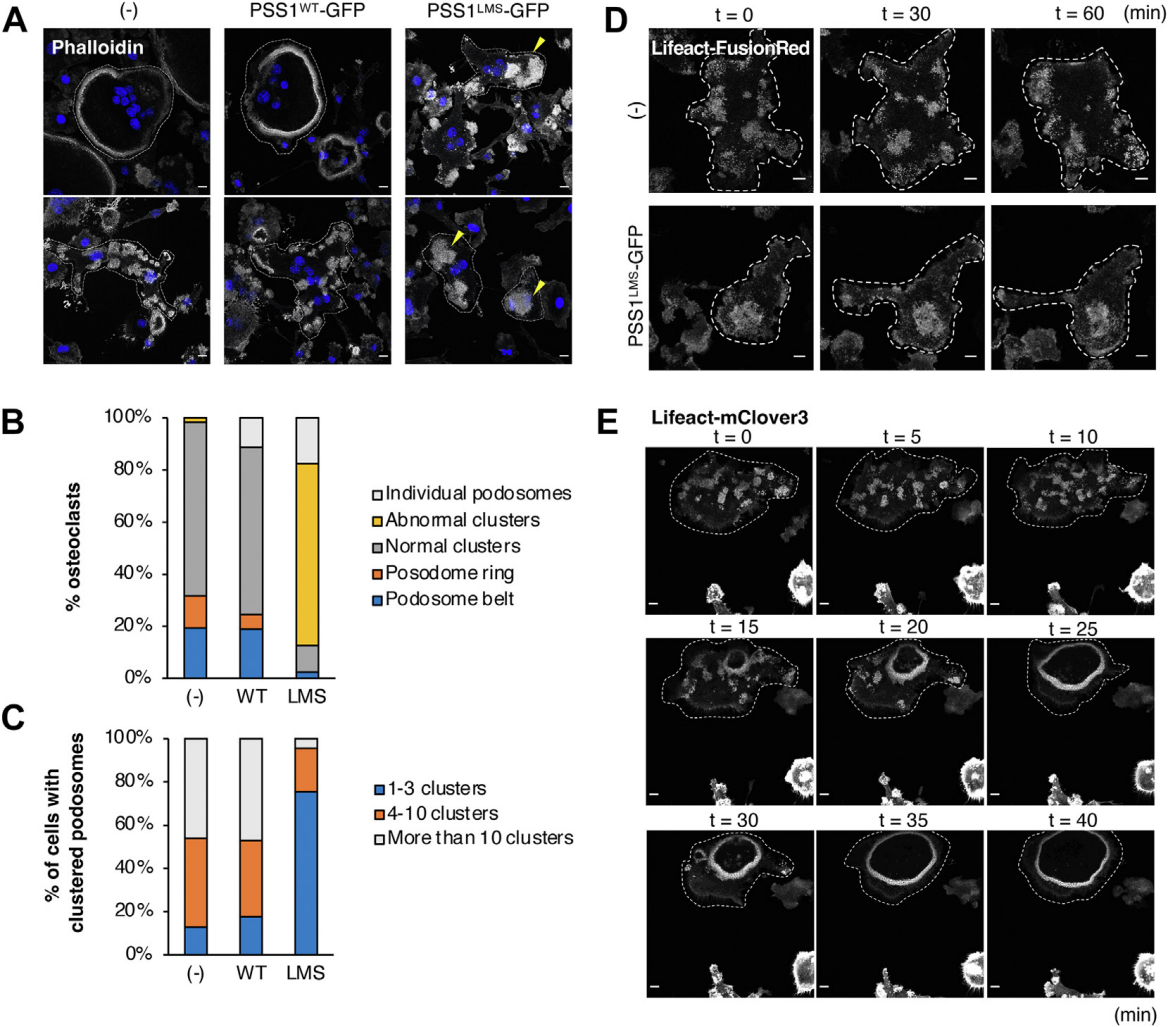
**PSS1**^**LMS**^**-expressing OCs show abnormal patterns of actin cytoskeleton**. A: Non-transduced, PSS1^WT^- or PSS1^LMS^-expressing OCs were stained with phalloidin. Images of OCs with podosome belts (top) or clusters (bottom) were shown for (−) and PSS1^WT^-GFP. Images of OCs with abnormal podosome clusters were shown for PSS1^LMS^-GFP. Scale bars, 10 μm. Yellow arrowhead, abnormal clusters. Dot lines outline OCs. B: OCs with individual or clustered podosomes were classified by their actin patterns and counted. Total of 39 (non-transduced), 40 (WT), and 84 (LMS) cells were counted from 4 individual experiments. C: The number of podosome clusters per OC was counted in non-transduced, PSS1^WT^- or PSS1^LMS^-expressing OCs. A total of 39 (non-transduced), 34 (WT), and 69 (LMS) cells were counted from 4 individual experiments. D: Lifeact-FusionRed was co-expressed with PSS1 in OCs, and live imaging was conducted. Scale bars, 10 μm. Dot lines outline OCs. E: An OC expressing Lifeact-mClover3 was live-imaged. Dot lines outline the OC. Scale bars, 10 μm.

To see if the actin cytoskeleton was altered by PSS1^LMS^, we observed actin in OCs with fluorescent phalloidin using confocal microscopy. Some of the non-transduced cells or PSS1^WT^-expressing cells had podosome belts (Fig. 2A) while others had many small clusters of podosomes (Fig. 2A–C). In contrast, the PSS1^LMS^-expressing cells had no podosome belts but instead had abnormal podosome clusters (Fig. 2A), which were larger in size and fewer in number than the podosome clusters in the other cells (Fig. 2B, C). We live-imaged these clusters by expressing Lifeact-FusionRed in the OCs. In non-transduced cells, podosome clusters repeatedly appeared and disappeared, dynamically changing the cells’ actin cytoskeleton. However, in PSS1^LMS^-expressing cells, podosomes did form but remained near the formation site, implying that PSS1^LMS^ causes defects in the dynamics of podosome clusters (Fig. 2D, supplemental Videos S1 and S2). Since the normal development of the actin cytoskeleton in OCs is essential for their multinucleation and bone resorption, this abnormality in the patterns and dynamics of podosome clusters may be one of the reasons for the decreased formation and activity of PSS1^LMS^-expressing OCs.

When we live-imaged a non-transduced cell during its change from podosome clusters to podosome belts, we observed no structures similar to the abnormal clusters in PSS1^LMS^-expressing cells (Fig. 2E). This suggests that PSS1^LMS^ inhibits the formation of correct patterns of podosome clusters, rather than inhibits the transformation from clusters to rings or belts.

Actin polymerization and depolymerization constantly occur within a podosome (32). To see if PSS1^LMS^ affects the turnover of actin, OCs expressing FusionRed-actin were analyzed by Fluorescence Recovery After Photobleaching (FRAP) (supplemental Fig. S4A). The velocity and percentage of fluorescence recovery were comparable between PSS1^WT^- and PSS1^LMS^-expressing cells (supplemental Fig. S4B). The localization of several actin-associated proteins that have important roles in actin turnover was also analyzed. Vinculin, paxillin, and talin are representative focal adhesion proteins, which localize to the clouds of podosomes in OCs. All of these proteins showed clear localization to the podosome cloud and comparable expression levels among non-transduced, PSS1^WT^- and PSS1^LMS^-expressing cells (supplemental Fig. S5). Together, these results suggest that PSS1^LMS^ does not affect actin turnover or the localization and amount of actin-associated proteins in OCs.

**Fig. 3:**
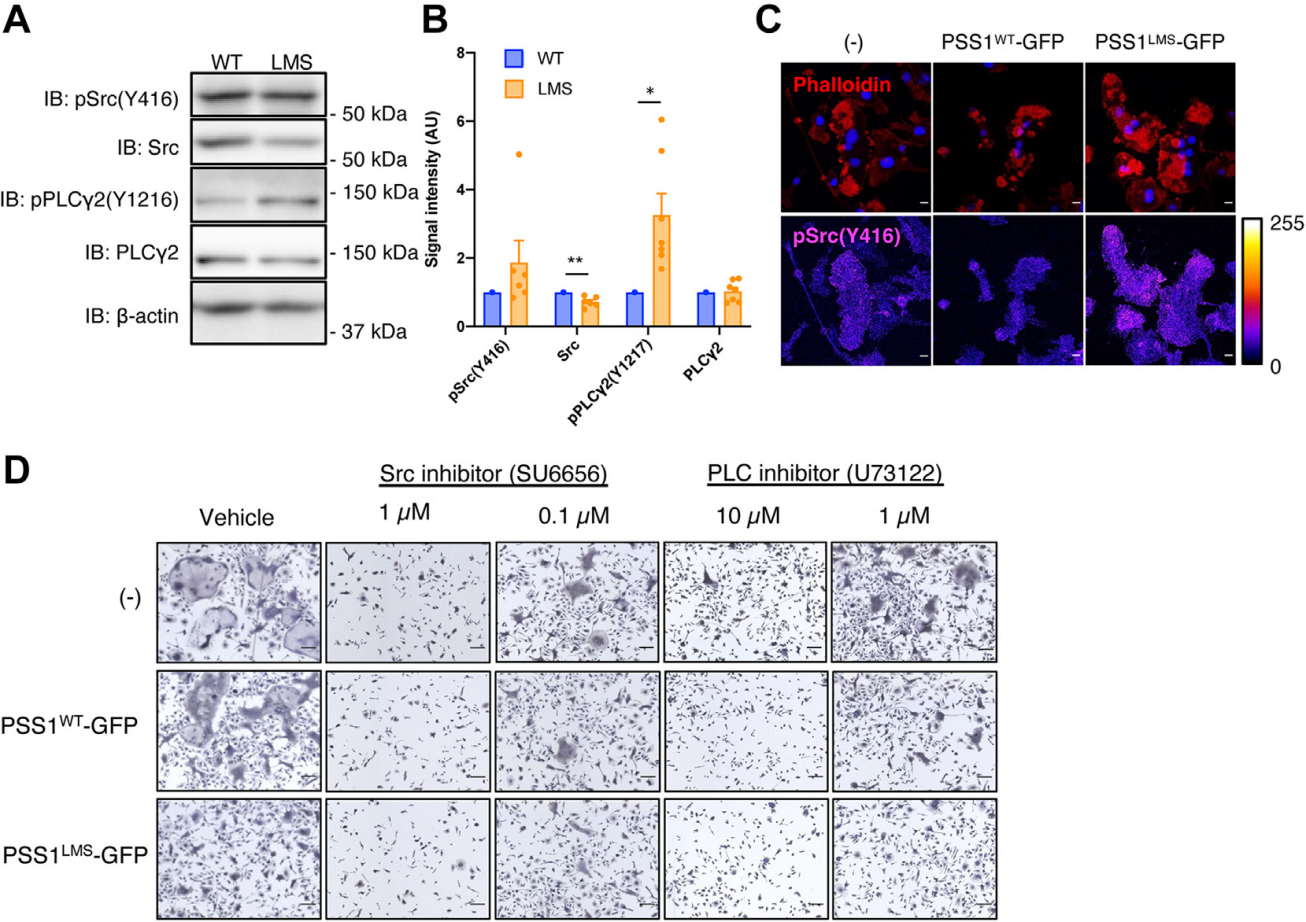
**Phosphorylationof Src and PLCγ2 are increased in PSS1**^**LMS**^**-expressing OCs**. A: PSS1^WT^- or PSS1^LMS^-GFP was expressed in OCs, and OCs were selected by puromycin. Phosphorylation levels of Src (Y416) and PLCγ2 (Y1217) were analyzed. B: The signal intensity in western blotting was measured by image J. The intensity in PSS1^WT^-GFP expressing samples was set as 1 in each experiment. Data are Mean + SEM (n = 7). ∗*P* < 0.05; ∗∗*P* < 0.01. Statistical analysis was done by a one-sample *t* test. C: Non-transduced, PSS1^WT^- or PSS1^LMS^-GFP-expressing OCs were stained with anti-pSrc (Y416) antibody and phalloidin. Scale bars, 10 μm. D: PSS1^WT^- or PSS1^LMS^-GFP-expressing OCs were treated with a Src or PLC inhibitor and OC formation was observed by TRAP staining. The inhibitors were added from day 3 to day 5. Scale bars, 100 μm.

Src and PLCγ2 are the two major regulators of actin cytoskeleton patterning in OCs (29, 33). To see if Src or PLCγ2 activity was suppressed or not in PSS1^LMS^-expressing cells, we analyzed the phosphorylation levels of Src at Y416 [pSrc (Y416)] and PLCγ2 at Y1217 [pPLCγ2 (Y1217)], the indicators of their activity, by western blotting. As a result, they were unchanged or increased in PSS1^LMS^-expressing cells (Fig. 3A, B). Since the total amount of Src decreased in PSS1^LMS^-expressing cells, the ratio of pSrc (Y416) to total Src was elevated. When we detected pSrc (Y416) by immunofluorescence, PSS1^LMS^-expressing cells showed an increased signal (Fig. 3C). Thus, Src and PLCγ2 activity was not decreased but rather increased in PSS1^LMS^-expressing cells. Furthermore, inhibitors of Src and PLCγ2 suppressed the OC formation in non-transduced, PSS1^WT^-, and PSS1^LMS^-expressing cells in a concentration-dependent manner (Fig. 3D), which indicates that increased Src and PLCγ2 activity is not the cause of the OC defects in PSS1^LMS^-expressing cells.

### PSS1^LMS^ changes membrane phospholipid composition

**Fig. 4:**
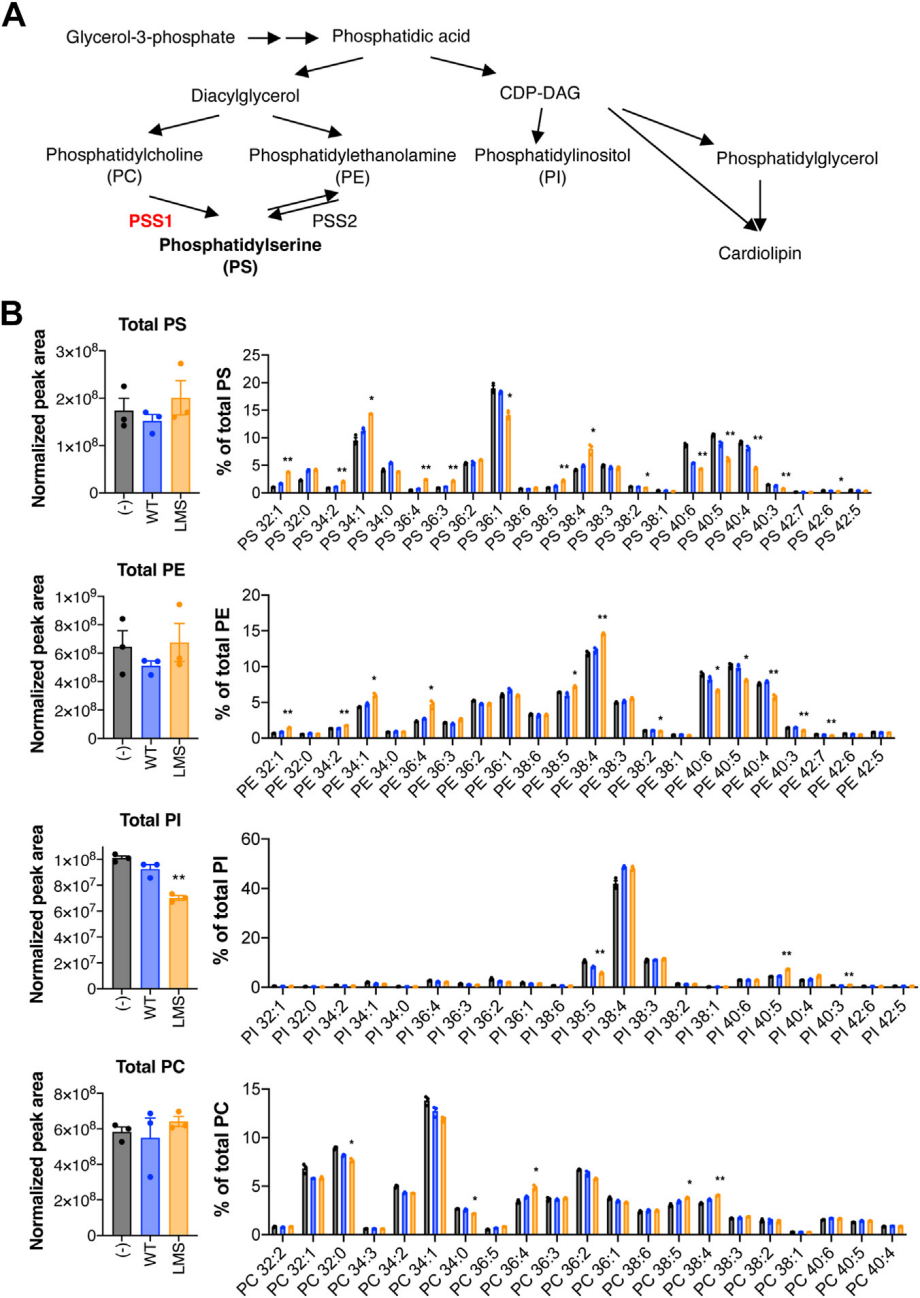
**Uncontrolled PS synthesis by PSS1**^**LMS**^**causes changes in phospholipid composition**. A: The phospholipid synthetic pathway. B: Phospholipids in non-transduced, PSS1^WT^- or PSS1^LMS^-expressing OCs were analyzed by LC-MS/MS. The normalized peak area of the total amount and compositions of molecular species are shown. Data are Mean + SEM (n = 3). ∗*P* < 0.05; ∗∗*P* < 0.01. ∗ is shown where non-transduced versus PSS1^LMS^-GFP and PSS1^WT^-GFP versus PSS1^LMS^-GFP were both significant. Statistical analysis was done by one-way ANOVA with Tukey’s test.

Next, we analyzed phospholipids of PSS1^WT^- or PSS1^LMS^-expressing OCs by LC-MS/MS (Fig. 4A, B). The total amounts of PS and PE were not significantly different between OCs expressing PSS1^WT^ and OCs expressing PSS1^LMS^, albeit PS and PE synthetic activities were increased by PSS1^LMS^ as previously reported in fibroblasts from patients with LMS (10). However, the compositions of PS and PE molecular species in PSS1^LMS^-expressing cells were different from those in non-transduced and PSS1^WT^-expressing cells. For example, in PSS1^LMS^-expressing cells, the levels of 32:1 (fatty acyl chains with 32 carbons and 1 double bond), 34:1, and 38:4 PS were higher than those in the non-transduced and PSS1^WT^-expressing cells, while the levels of 36:1, 40:5, and 40:4 PS were lower. Similar differences were observed for PE. Unexpectedly, PI, whose synthesis pathway is different from that of PS, was decreased without any change in its composition of molecular species. The amounts of PI monophosphate (PIP) and PI bisphosphate (PIP_2_) were also measured using supercritical fluid chromatography – MS/MS (24). Every PIP and PIP_2_ molecular species decreased regardless of their phosphorylation patterns (supplemental Fig. S6). For PC, the amount and molecular species in PSS1^LMS^-expressing cells differed little from those in the non-transduced and PSS1^WT^-expressing cells (Fig. 4B).

To see if PSS1^LMS^ expression affected de novo phospholipid synthesis, we generated a HeLa cell line stably expressing PSS1^LMS^ (supplemental Fig. S7A) and cultured them in the presence of [^14^C]glycerol or [^32^P]orthophosphate. The incorporation of the radioisotopes into PC, PE, and PS was higher in PSS1^LMS^-expressing cells than in non-transduced and PSS1^WT^-expressing cells (supplemental Fig. S7B–D). However, the incorporation of the radioisotopes into PI was comparable among non-transduced, PSS1^WT^- and PSS1^LMS^-expressing cells, suggesting that PI synthesis was relatively suppressed in PSS1^LMS^-expressing cells. We also examined total phospholipid degradation with a pulse-chase experiment using [^14^C]glycerol (supplemental Fig. S7E). Although more [^14^C]glycerol was incorporated into phospholipids in PSS1^LMS^-expressing cells than in PSS1^WT^-expressing cells at 0 h of the chase, the radiolabeled phospholipids degraded more rapidly in PSS1^LMS^-expressing cells than in PSS1^WT^-expressing cells.

Sohn *et al.* have shown that overexpression of PSS1^LMS^ causes PS accumulation in the ER in HEK293T cells (34). When OCs were stained with recombinant PS probe 2xPH (evt2)-His (22, 35), the signal was detected in the plasma membrane and perinuclear region, which presumably means the perinuclear signal was coming from the Golgi and recycling endosomes (supplemental Fig. S8). Neither PSS1^WT^-GFP nor PSS1^LMS^-GFP expression affected the 2xPH (evt2)-His staining pattern, and the signal of 2xPH (evt2)-His was separated from that of PSS1-GFP, which localized to ER and nuclear membrane.

### Introducing an inactive mutation into PSS1^LMS^ canceled the effects of PSS1^LMS^ on OCs

It remains unclear if the uncontrolled synthesis of PS by PSS1^LMS^ is the cause of the LMS phenotypes. To address this issue in the OC system, we mutated the amino acid residues crucial for PSS1’s catalytic reaction. Eight mutations of PSS1 were previously shown to inactivate the enzyme (36). Of these, we chose the E200A mutation and confirmed that additional mutation of E200A in PSS1^LMS^ suppressed PS synthesis (supplemental Fig. S9A, B).

**Fig. 5:**
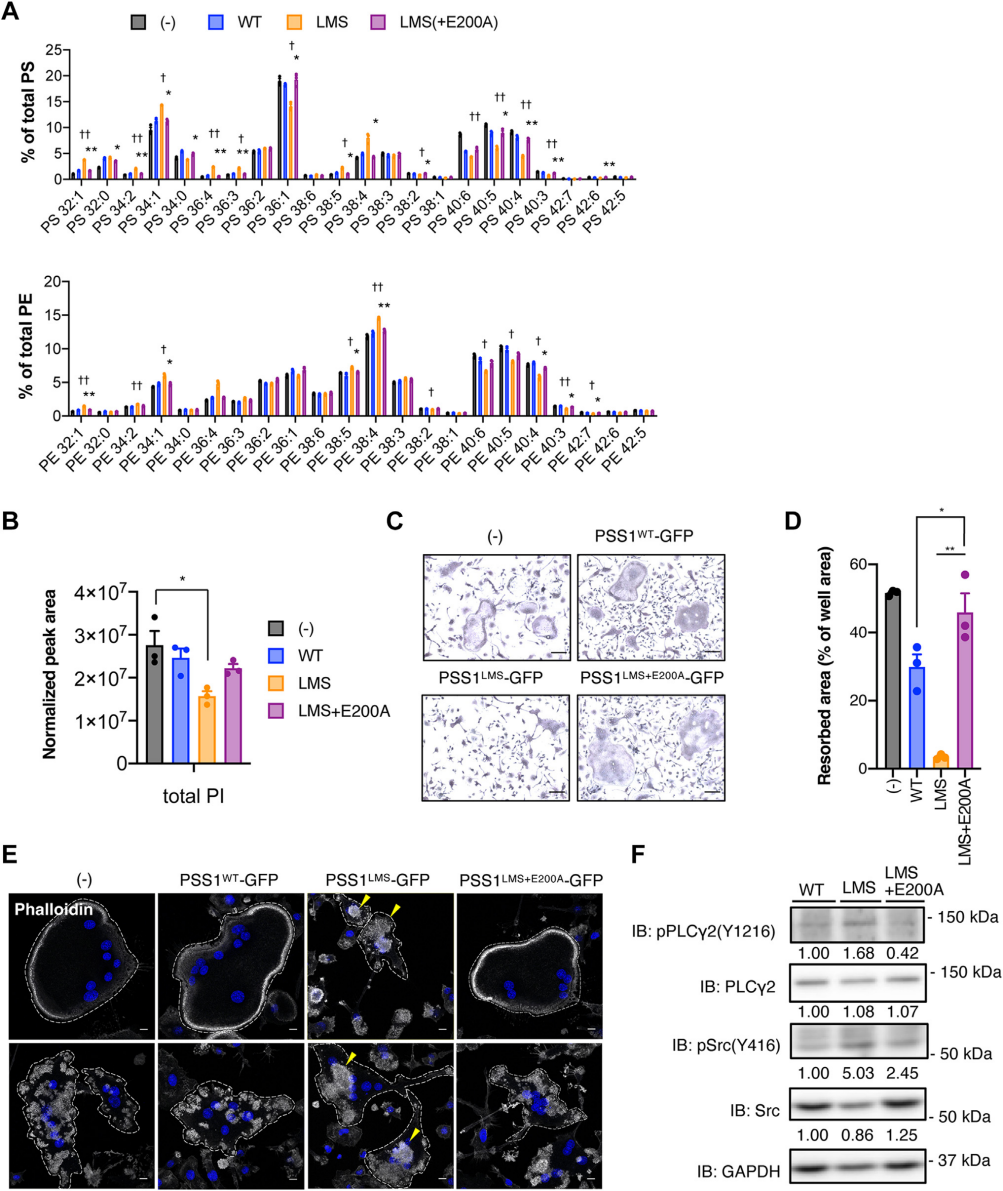
**Introducing an inactive mutation into PSS1**^**LMS**^**results in no effects on OCs**. A: The composition of molecular species of PS and PE were analyzed by LC-MS/MS. Data are Mean ± SEM (n = 3). ∗ or †, *P* < 0.05; ∗∗ or ††, *P* < 0.01. Statistical analysis was done by one-way ANOVA with Tukey’s test. ∗ is shown where LMS and LMS+E200A were compared. † is shown where LMS was significantly different from both (−) and WT. B: The total peak area of PI in LC-MS/MS analysis is shown. Data are Mean + SEM (n = 3). ∗*P* < 0.05. Statistical analysis was done by one-way ANOVA with Tukey’s test. C: PSS1-expressing OCs were detected by TRAP staining. Scale bars, 100 μm. D: PSS1-expressing OCs were cultured in calcium phosphate-coated plates, and degraded pits were measured with ImageJ. Data are Mean ± SEM (n = 3). Statistical analysis was done by one-way ANOVA with Tukey’s test. E: PSS1^WT^-, PSS1^LMS^- or PSS1^LMS+E200A^-expressing OCs were stained with phalloidin. Yellow arrowheads indicate abnormal clusters of podosomes. Scale bars, 10 μm. F: PSS1^WT^-GFP, PSS1^LMS^-GFP, or PSS1^LMS+E200A^-GFP was expressed in OCs, and OCs were selected by puromycin. The phosphorylation levels of Src (Y416) and PLCγ2 (Y1217) were analyzed. The signal intensity was measured by ImageJ and shown in an arbitrary unit.

The PS and PE molecular species (Fig. 5A) and PI amount (Fig. 5B) in PSS1^LMS+E200A^-expressing OCs were not different from those in the non-transduced cells, indicating that the changes in phospholipid composition in PSS1^LMS^-expressing cells are due to uncontrolled PS synthesis by PSS1^LMS^.

Finally, we examined whether the expression of PSS1^LMS+E200A^ caused the same OC phenotypes as PSS1^LMS^. In the TRAP staining and the extracellular acidification assay, PSS1^LMS+E200A^ had no inhibitory effect on the formation or activity of OCs (Fig. 5C, D). PSS1^LMS+E200A^ also did not affect podosome cluster patterns, podosome belt formation (Fig. 5E), or the levels of pSrc (Y416) or pPLCγ2 (Y1217) (Fig. 5F). Together, these results support the hypothesis that uncontrolled PS synthesis itself causes the defects in actin belt formation, multinucleation, and activity of OCs.

### A gain-of-function mutant of PSS2 inhibits the formation of OCs

**Fig. 6:**
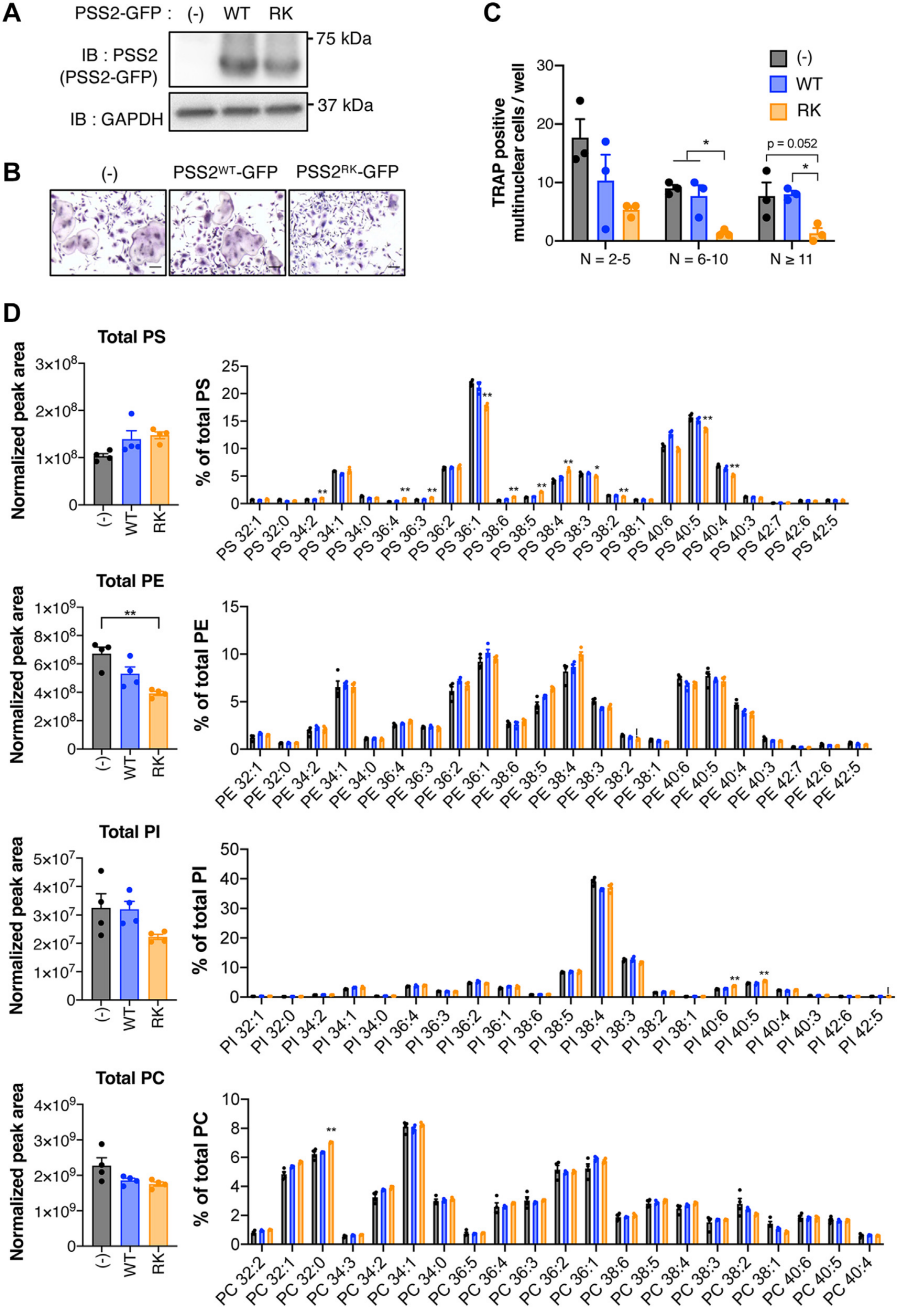
**A gain-of-function mutant of PSS2 causes impaired OC formation and altered phospholipid composition**. A: GFP-fusion protein of wild type PSS2 (PSS2^WT^-GFP) or PSS2 R97K mutant (PSS2^RK^-GFP) was expressed in OCs, and OCs were selected by puromycin. The amount of PSS2 was analyzed by immunoblotting. GAPDH was used as a loading control. B: Non-transduced, PSS2^WT^- or PSS2^RK^-expressing OCs were detected by TRAP staining. Scale bars, 100 μm. C: TRAP-positive multinucleated OCs in (B) were counted. N, number of nuclei per cell. Data are Mean ± SEM (n = 3). ∗*P* < 0.05. Statistical analysis was done by one-way ANOVA with Tukey’s test. D: Phospholipids in non-transduced, PSS2^WT^- or PSS2^RK^-expressing OCs were analyzed by LC-MS/MS. The normalized peak area of the total amount and compositions of molecular species are shown. Data are Mean + SEM (n = 4). ∗*P* < 0.05; ∗∗*P* < 0.01. ∗ is shown where non-transduced versus PSS1^LMS^-GFP and PSS1^WT^-GFP versus PSS1^LMS^-GFP were both significant. Statistical analysis was done by one-way ANOVA with Tukey’s test.

Although no mutations in *PTDSS2* have been found in LMS, the R97K mutant of rodent PSS2 (PSS2^RK^) has been reported to be resistant to feedback inhibition by PS (7). Since PSS2 synthesizes PS from PE and the fatty acid compositions of PC and PE are different, the effect of PSS2^RK^ on phospholipid composition is expected to be different from that of PSS1^LMS^. Therefore, to further investigate the relationship between changes in phospholipid composition and OC formation, we characterized OCs expressing wild-type PSS2 (PSS2^WT^) or PSS2^RK^. PSS2^WT^-expressing OCs exhibited multinucleated osteoclast formation, as did non-transduced OCs (Fig. 6A–C). However, PSS2^RK^-expressing OCs failed to form multinuclear OCs. A lipidomic analysis revealed that similar to PSS1^LMS^, PSS2^RK^ changed the composition of PS molecular species without affecting the total amount (Fig. 6D). For example, in PSS2^RK^-expressing OCs, the levels of 34:2, 36:4, and 38:4 PS were higher than those in the non-transduced and PSS1^WT^-expressing cells, while the levels of 36:1, 40:5, and 40:4 PS were lower. The total amount of PI also tended to decrease without much change in the molecular species. Although PSS2^RK^ did not affect the composition of PE molecular species, the total amount of PE tended to decrease. Thus, PSS2^RK^ impaired OC formation and caused changes in phospholipid composition, most of which were common in PSS1^LMS^.

## Discussion

In this study, we generated OCs expressing PSS1^LMS^ protein at nearly the same protein levels of PSS1^LMS^ as the levels of endogenous PSS1, a situation similar to that in LMS patients having a heterozygous dominant mutation in the *PTDSS1* gene. The PSS1^LMS^-expressing OCs had high PS synthetic activity comparable to that in LMS patient fibroblasts (10). Using these cells, we demonstrated that PSS1^LMS^ impeded the formation, multinucleation, and extracellular acidification activity of OCs. Clinically, defects in OCs appear to lead to osteopetrosis or osteosclerosis, in which bone density increases, and normally the bones become hard but fragile (18). For example, individuals with osteopetrosis carrying mutations in *TNFSF11* (encoding RANKL) were prone to bone fractures and their bone biopsy specimens lacked osteoclasts (37). Patients with osteopetrosis have a variety of symptoms (18), several of which are observed in patients with LMS, such as nasal airway obstruction and intellectual disability. These similarities in symptoms, together with the present results suggest that OC impairment is one of the causes of the symptoms in patients with LMS.

Although PSS1^LMS^ expression in OCs increased cellular PS and PE synthesis, it did not change the steady state levels of these phospholipids. This is probably due to the increases in phospholipid catabolism since PSS1^LMS^ expression increased the phospholipid degradation as well as the synthesis of PS, PE, and PC in HeLa cells. On the other hand, PSS1^LMS^ greatly affected the acyl chain compositions of PS and PE. In PSS1^LMS^-expressing cells, 34:1 PS increased and became the most dominant molecular species of PS. This might be due to the predominant synthesis of 34:1 PS from 34:1 PC (the major molecular species of PC) by PSS1^LMS^. However, other increased molecular species, such as 32:1, 32:0, 36:4, and 38:4 PS, were not especially abundant in PC. A specific portion of PC molecular species might be used in PS synthesis by PSS1. PE is synthesized by the both CDP-ethanolamine pathway and the PS decarboxylation pathway. Ethanolamine phosphotransferase 1 (EPT1), which is a major enzyme in the CDP-ethanolamine pathway, contributes to the synthesis of PE species such as 36:1, 36:4, 38:5, 38:4, 38:3, 40:6, 40:5, and 40:4 PE (38). Except for 38:4 PE, these molecular species were not increased in PSS1^LMS^-expressing cells. On the other hand, the changes in the acyl chain composition of PE were similar to those of PS. Thus, the altered acyl chain composition of PE is probably due to the conversion of PS into PE by PS decarboxylase. We also showed that the total amount of PI decreased by PSS1^LMS^ expression. A mutant CHO cell line carrying an R95K mutation in *Ptdss1* was reported to exhibit an increased PS synthetic activity and a reduced level of PI (6). A possible cause of the decreased level of PI is inhibition of PI synthesis. By measuring [^14^C]glycerol or [^32^P]orthophosphate incorporation into phospholipids, we found that PI synthesis relative to total phospholipid synthesis was decreased in PSS1^LMS^-expressing HeLa cells compared to parental or PSS1^WT^-expressing HeLa cells. This is in agreement with previous reports that the synthesis and amount of PI increased in a cell line defective in PS synthesis (39, 40). Together, these observations raise the possibility that the acyl chain composition of PS and PE have regulatory roles in the synthesis and level of PI.

RANKL-mediated gene induction is essential for the differentiation and fusion of OCs. However, PSS^LMS^ did not significantly affect the expression of these genes. Instead, PSS1^LMS^ caused abnormal podosome clusters with reduced dynamics and inhibited the normal development of podosome belts. The spatio-temporal dynamics of podosome clusters are important for the migration and fusion of OCs (29, 31). Furthermore, the structure of podosome belts is similar to that of sealing zones, which are critical to the bone-resorption function of OCs (30). Mice with defective podosome organization in OCs showed symptoms of osteopetrosis (41), suggesting that defects in podosome organization in OCs do result in in vivo phenotypes of increasing bone density. Src and PLCγ2 are two major regulators of podosome organization in OCs (29, 33). OCs deficient in these proteins had large abnormal podosome clusters (33, 41), which are quite similar to those observed in PSS1^LMS^-expressing OCs. However, since the phosphorylation levels of these proteins were not suppressed in PSS1^LMS^-expressing cells, the impaired actin podosome organization cannot be explained by the reduced function of these proteins.

Since the actin cytoskeleton forms in the proximity of membranes, many actin-associated proteins have phospholipid-interacting abilities or domains. Among the phospholipids that are affected in PSS1^LMS^-expressing cells, 36:1 PS has been shown to be involved in nanoclustering of GPI-anchored proteins in the plasma membrane in concert with the actin cytoskeleton (42). Nanoclustering of GPI-anchored proteins is dependent on the long acyl chains of PS in the inner leaflet that can interdigitate with the long acyl chains of GPI-anchored proteins in the outer leaflet, creating liquid order-like domains in the plasma membrane. The decrease in the level of 36:1 PS and the concomitant increase in the levels of PS with shorter acyl chains such as 34:1 PS might impair the organization of actin in PSS1^LMS^-expressing cells. PI phosphates (PIPs), the phosphorylated derivatives of PI, are explicitly recognized by a number of actin-associated proteins (43). PI and PIPs were decreased in PSS1^LMS^-expressing OCs, but we didn’t observe any differences in actin turnover or localization of vinculin, paxillin, and talin. Although PE is less known to interact with actin-associated proteins than PS and PIPs, PE containing long polyunsaturated fatty acids was shown to facilitate OC fusion, possibly by increasing membrane fluidity (44). PEs with polyunsaturated fatty acids, such as 40:6, 40:5, and 40:4 PE were also decreased in PSS1^LMS^-expressing OCs. Non-apoptotic PS exposure on the cell surface is also required for OC fusion (45). However, it is unlikely that impaired multinucleation in PSS1^LMS^-expressing OCs is due to impaired PS exposure because PSS1^LMS^ increases PS levels on the cell surface (10). Interestingly, PSS2^RK^, a gain-of-function mutant of PSS2, also impeded OC formation and caused changes in phospholipid composition similar to those caused by PSS1^LMS^. These results support the hypothesis that uncontrolled PS synthesis by PSS1^LMS^ causes changes in fatty acid composition and amount in certain phospholipid classes, resulting in impaired OC formation and function. However, the changes in phospholipids induced by PSS1^LMS^ and PSS2^RK^ were so similar that we could not further narrow down the phospholipid species that impair OC formation and function. Further experiments are needed to identify the phospholipid species responsible for the abnormal podosome clusters or impaired multinucleation of OCs.

In summary, we showed that PSS1^LMS^-expressing OCs had reduced podosome belt formation, multinucleation, and activity. Uncontrolled PS synthesis caused various changes in the amount and acyl chain composition of phospholipids, which appeared to dysregulate the pattern formation of the actin cytoskeleton in OCs. Since the dynamics of the actin cytoskeleton pattern is an essential process for OC function, it might be the cause of the reduced formation and activity of OCs. The present results suggest that altering the phospholipid profiles of OCs impairs their formation and function, which might be one of the causes of progressive osteosclerosis in patients with LMS.

## Data Availability

The microarray data can be accessed at the Gene Expression Omnibus repository (GSE207351). The datasets generated and analyzed during this study are available from the corresponding author on reasonable request.

## Supplemental data

This article contains supplemental data.

## Conflict of interest

The authors declare that they have no known competing financial interests or personal relationships that could have appeared to influence the work reported in this paper.
