# Supplementary material for: Disease-related PSS1 mutant impedes the formation and function of osteoclasts
Source: J Lipid Res. 2023 Sep 14;64(11):100443. doi: 10.1016/j.jlr.2023.100443 (PMC10641532; doi:10.1016/j.jlr.2023.100443)
Supplement: Supplemental data [file mmc1.pdf]

Supplemental Fig. S1

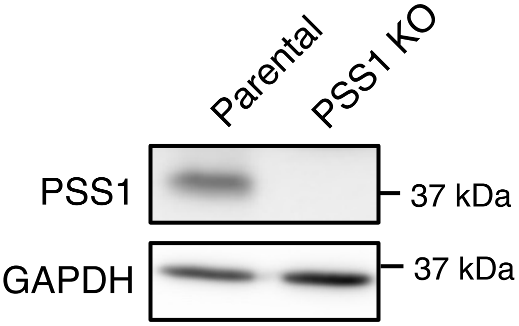

Supplemental Fig. S1. Validation of PSS1 antibody.

Total cell lysates from parental HeLa cells and PSS1 KO HeLa cells were immunoblotted with anti-PSS1 antibody and anti-GAPDH antibody.

Supplemental Fig. S2

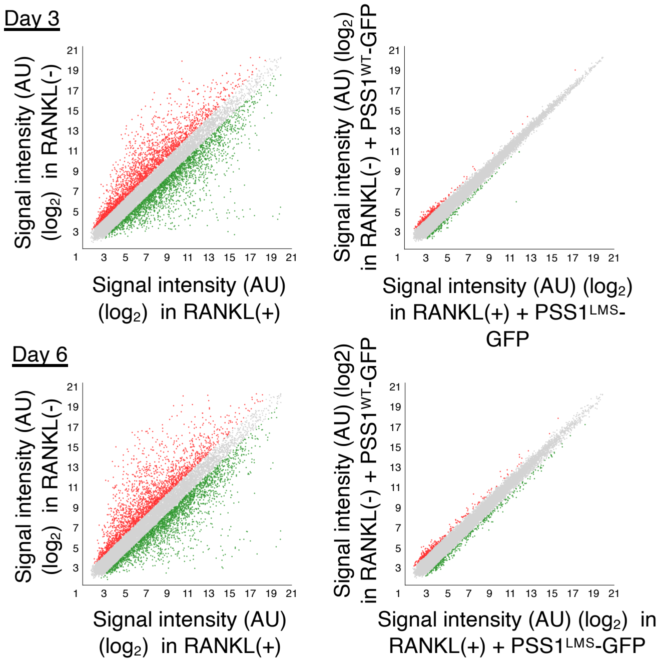

Supplemental Fig. S2. PSS1^{LMS} does not affect gene expression during OC differentiation. Scatter plots comparing gene expression patterns in RANKL-untreated or -treated samples, and in RANKL-treated PSS1^{WT}- or PSS1^{LMS}-expressing samples, at day 3 or 6. AU, arbitrary unit.

Supplemental Fig. S3

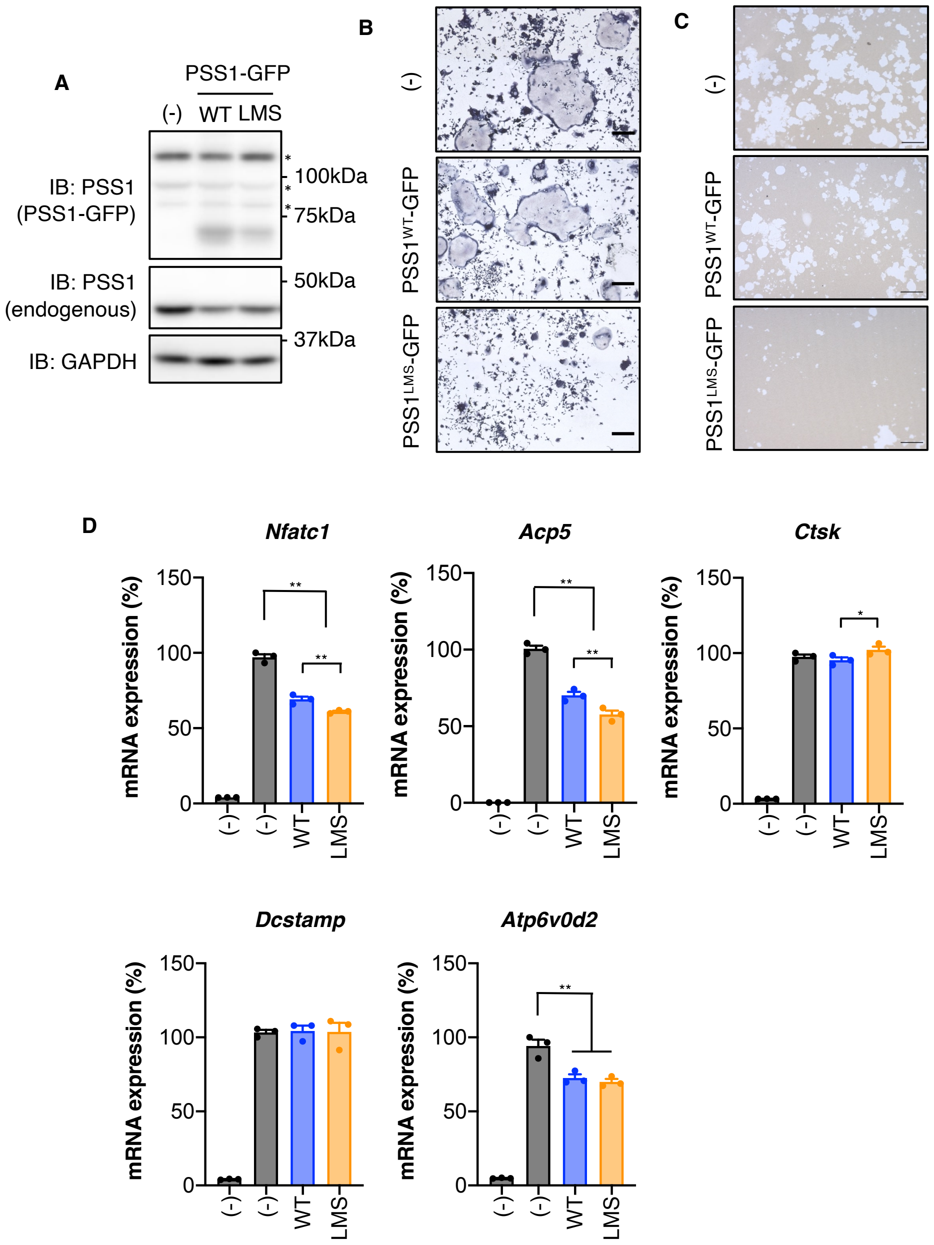

Supplemental Fig. S3. RAW264.7 stably expressing PSS1^{LMS}-GFP shows defects in OC multinucleation and activity.

(A) PSS1-GFP expression was confirmed by western blotting. *, nonspecific bands. (B) RAW264.7 cells stably expressing PSS1^{WT} or PSS1^{LMS} were treated with MCSF and RANKL and detected with TRAP staining. Scale bars, 200 μm. (C) RAW264.7 cells stably expressing PSS1^{WT} or PSS1^{LMS} were treated with MCSF and RANKL on calcium-phosphate coated wells. The degraded pits were imaged. Scale bars, 200 μm. (D) The expression of OC genes was analyzed by qRT-PCR and normalized to β-actin mRNA expression. Data are Mean + SEM (n = 3). * p<0.05; **, p <0.01. Statistical analysis was done by one-way ANOVA with Tukey's test.

Supplemental Fig. S4

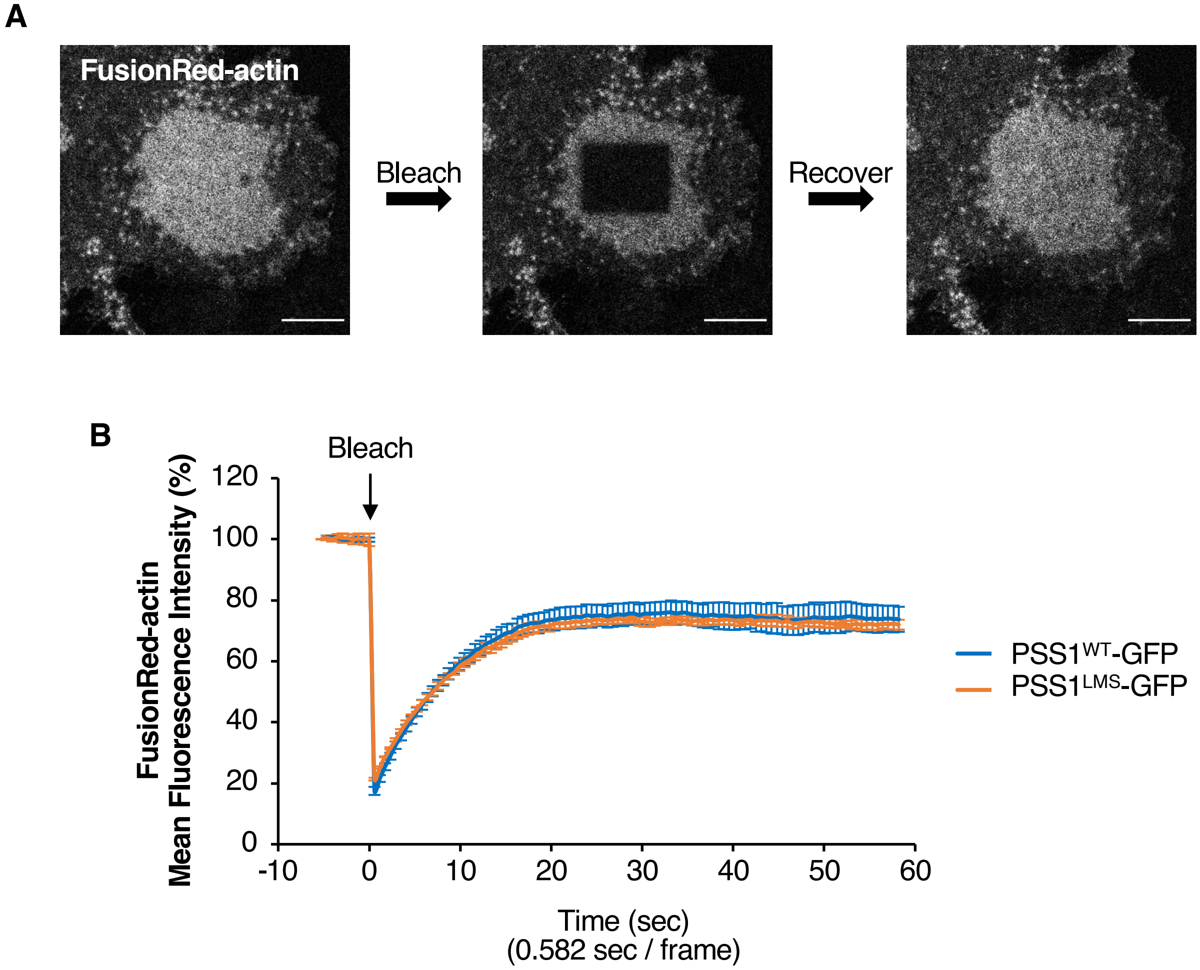

Supplemental Fig. S4. Actin turnover is not affected in PSS1^{LMS}-expressing OCs.

(A) A scheme of FRAP (Fluorescence recovery after photobleaching) experiments. The image is a cluster of podosomes in an OC. Scale bars, 10 µm. (B) PSS1^{WT}-GFP or PSS1^{LMS}-GFP was co-expressed in OCs with FusionRed-actin. Mean fluorescence intensity of FusionRed-actin was quantified in the bleached area and plotted. n=3-5.

Supplemental Fig. S5

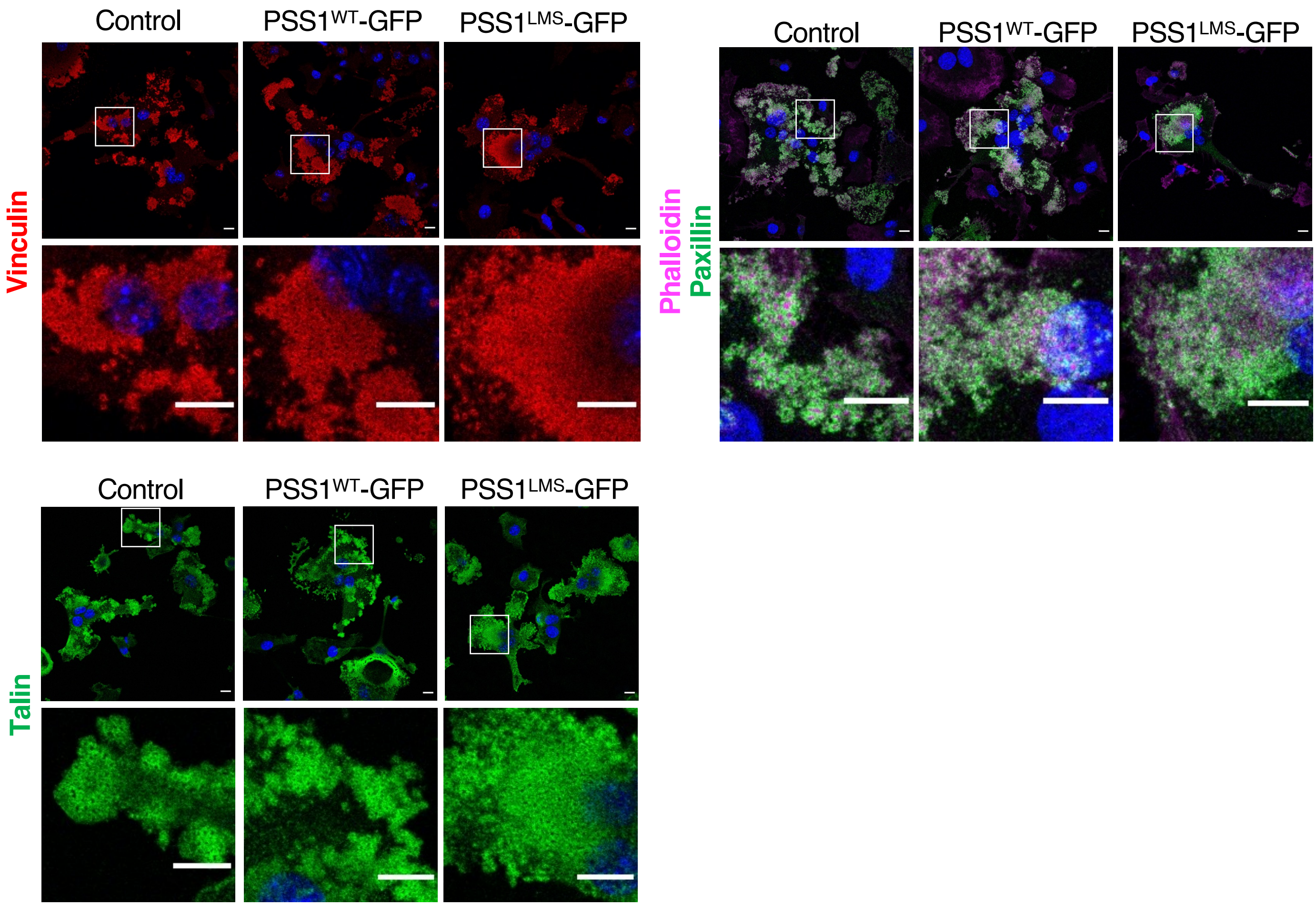

Supplemental Fig. S5. The localization of actin-associated proteins is not affected in PSS1^{LMS} expressing OCs

PSS1^{WT}-GFP or PSS1^{LMS}-GFP was expressed in OCs. The OCs were stained for vinculin, paxillin, talin and phalloidin. Scale bars, 10 μm.

Supplemental Fig. S6

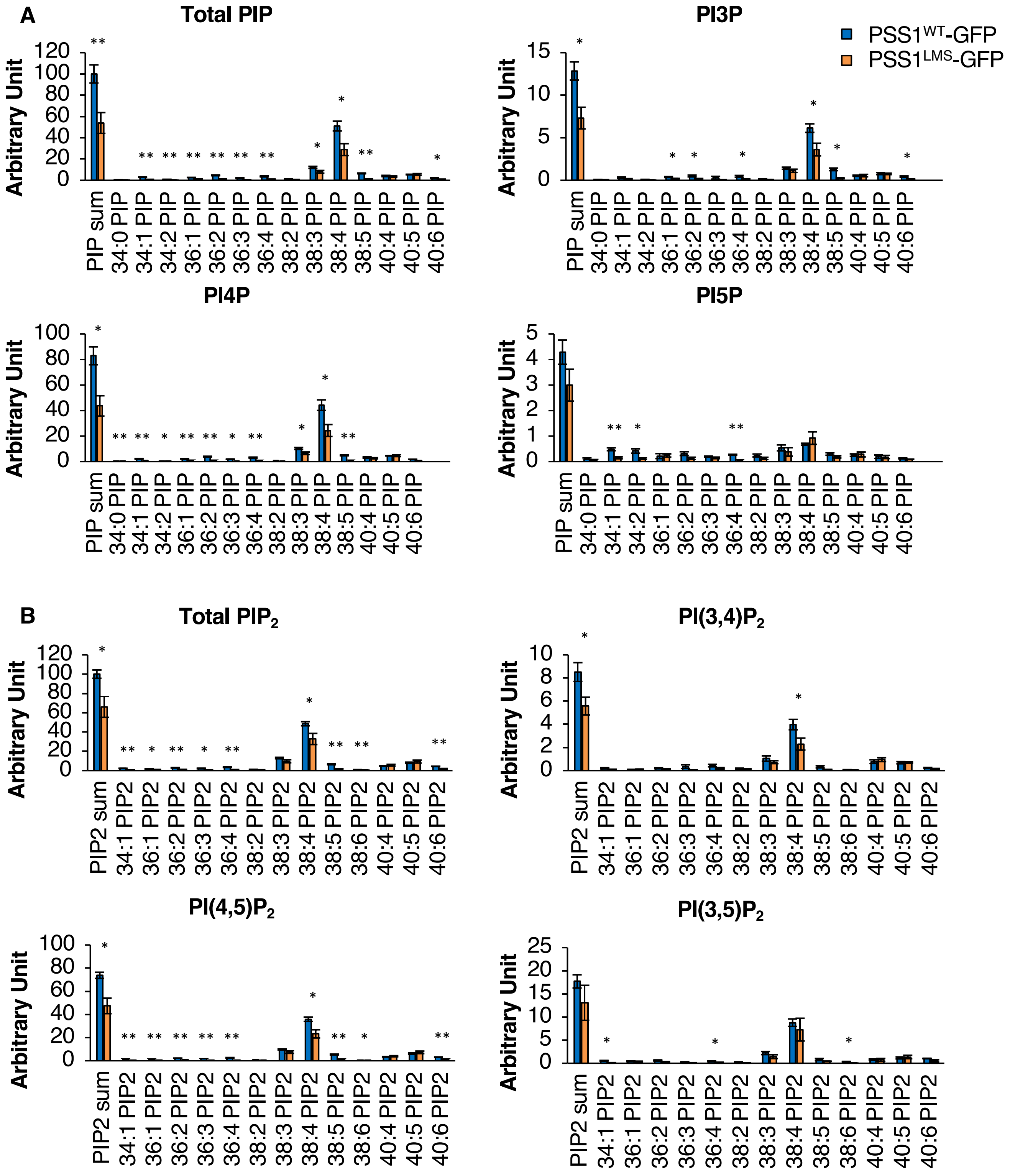

Supplemental Fig. S6. $PSS1^{LMS}$ induces decrease in the amount of PIP and PIP_2

The amounts of PIP(A) and PIP_2(B) were analyzed by SFC-MS/MS. PIP and PIP_2 signals were normalized by 36:2 PS, the amount of which was not affected by $PSS1^{LMS}$ expression. Mean ± SEM. n=4. **p*<0.05; ***p*<0.01. Statistical analysis was done by Student's *t*-test.

Supplemental Fig. S7

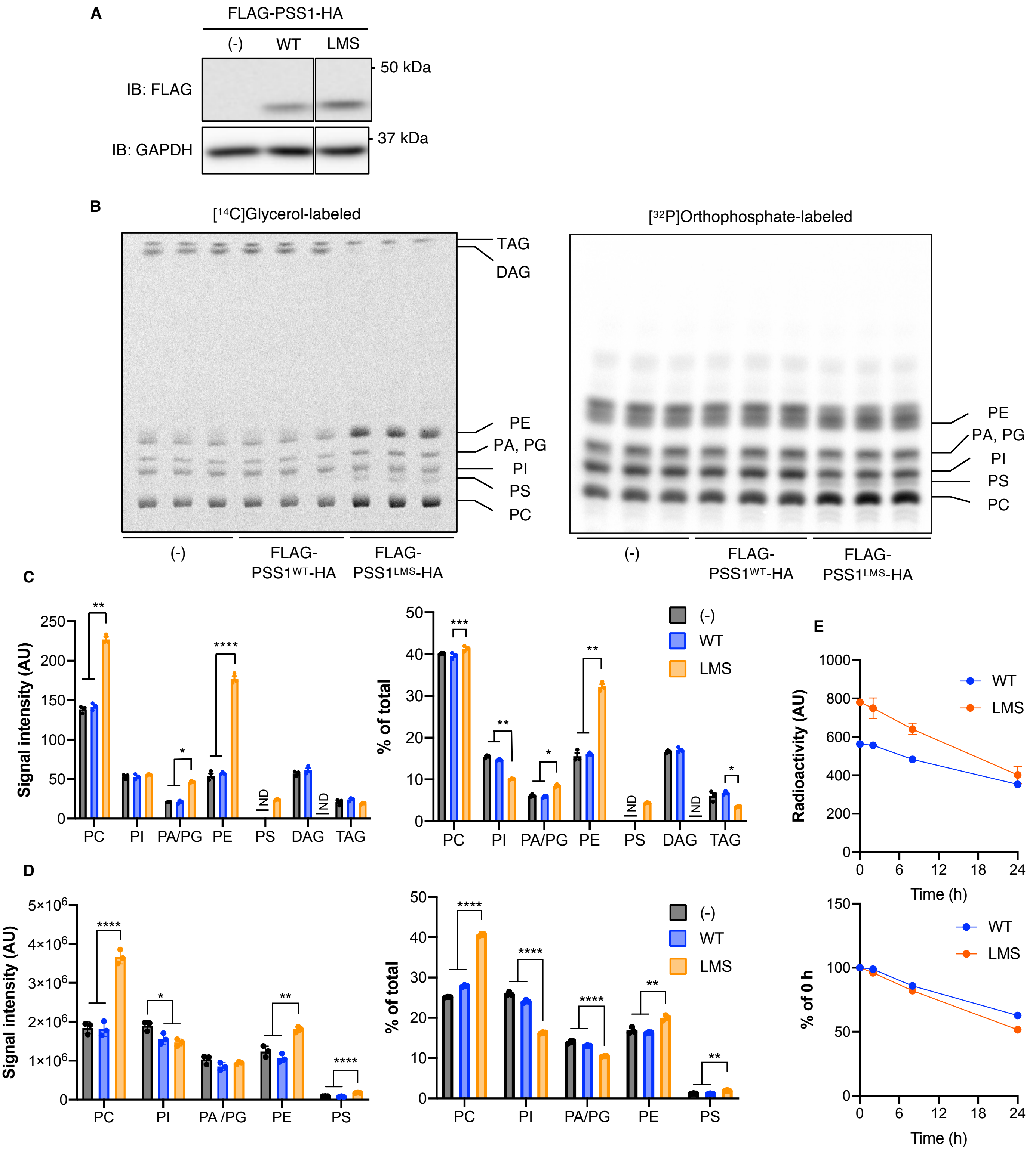

Supplemental Fig. S7. PI synthesis relative to total phospholipid synthesis is decreased in PSS1LMS-expressing HeLa cells

(A) FLAG-PSS1WT-HA or FLAG-PSS1LMS-HA was stably expressed in HeLa cells. Expressed proteins were detected with anti-FLAG antibody. (-), WT, and LMS indicate non-transduced cells, FLAG-PSS1WT-HA expressing cells, and FLAG-PSS1LMS-HA cells, respectively. (B) HeLa cells were labeled with [^{14}C]glycerol or [^{32}P]orthophosphate. Incorporated [^{14}C]glycerol or [^{32}P]orthophosphates in phospholipid was analyzed by thin-layer chromatography. (C, D) Radioactivity signal of [^{14}C]glycerol (C) or [^{32}P]orthophosphates (D) was analyzed by ImageJ. Data are Mean + SEM (n = 3). Statistical analysis was done by one-way ANOVA with Tukey's test. (E) HeLa cells were labeled with [^{14}C]glycerol for 12 hours. After washout, incorporated [^{14}C]glycerol in phospholipid was chased for 0, 2, 8, or 24 hours. Data are Mean ± SD (n = 2).

Supplemental Fig. S8

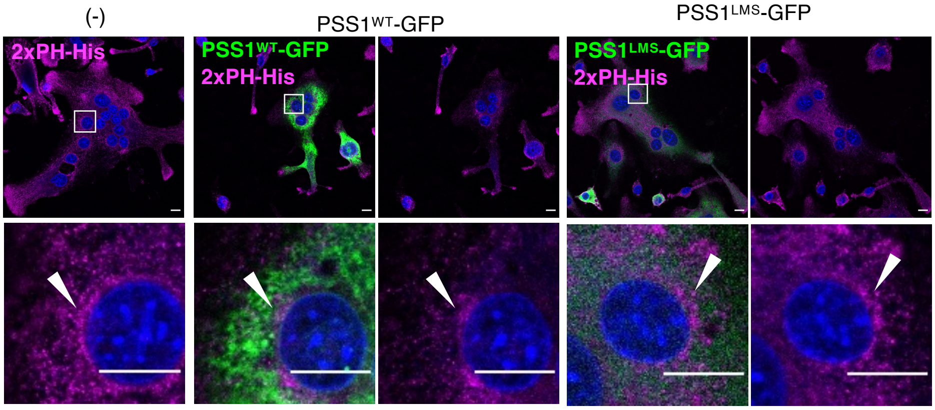

Supplemental Fig. S8. PS localization in PSS1^{LMS}-expressing cells
PSS1^{LMS} expressing OCs were stained with a recombinant PS probe 2xPH-His. Arrowhead, perinuclear staining of OCs. Scale bars, 10 µm.

Supplemental Fig. S9

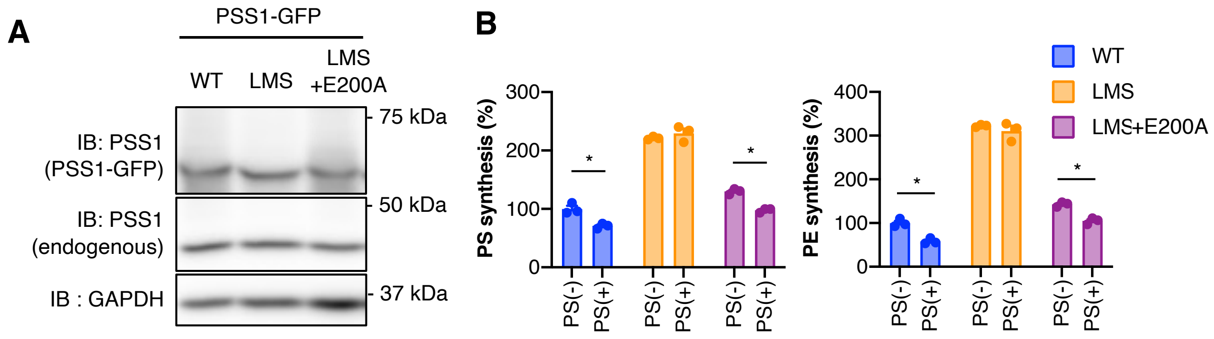

Supplemental Fig. S9. Introduction of inactive mutation into PSS1^{LMS} suppresses PS synthesis

(A) PSS1^{WT}-GFP, PSS1^{LMS}-GFP, or PSS1$^{LMS+E200A}$-GFP was expressed in OCs, and OCs were selected by puromycin. The data is from the same experiment as in Fig. 1A. (B) PS and PE synthesis in PSS1^{WT}-, PSS1^{LMS}- or PSS1$^{LMS+E200A}$-expressing OCs were measured by [^{14}C]serine incorporation, with or without exogenous 20 µM brain PS. Data are Mean + SEM (n = 3). The data of PSS1^{WT}- and PSS1^{LMS}-expressing OCs are the same as Fig. 1B.

Legends to Supplemental Videos

Supplemental Video S1

The dynamics of podosome clusters in osteoclasts.

Osteoclasts transduced with Lifeact-FusionRed were live-imaged for 60 minutes with confocal microscopy. Images were taken every minute. Scale bar, 10 µm.

Supplemental Video S2

The dynamics of podosome clusters in osteoclasts with PSS1^{LMS}-GFP.

Osteoclasts transduced with Lifeact-FusionRed and PSS1^{LMS}-GFP were live-imaged for 60 minutes with confocal microscopy. Images were taken every minute. Scale bar, 10 µm.
